# Dengue Early Warning System and Outbreak Prediction Tool in Bangladesh Using Interpretable Tree‐Based Machine Learning Model

**DOI:** 10.1002/hsr2.70726

**Published:** 2025-05-09

**Authors:** Md. Siddikur Rahman, Miftahuzzannat Amrin, Md. Abu Bokkor Shiddik

**Affiliations:** ^1^ Department of Statistics Begum Rokeya University Rangpur Bangladesh

**Keywords:** artificial intelligence, dengue, early warning, infectious disease, prediction

## Abstract

**Background and Aims:**

A life‐threatening vector‐borne disease, dengue fever (DF), poses significant global public health and economic threats, including Bangladesh. Determining dengue risk factors is crucial for early warning systems to forecast disease epidemics and develop efficient control strategies. To address this, we propose an interpretable tree‐based machine learning (ML) model for dengue early warning systems and outbreak prediction in Bangladesh based on climatic, sociodemographic, and landscape factors.

**Methods:**

A framework for forecasting DF risk was developed by using high‐performance ML algorithms, namely Random Forests, eXtreme Gradient Boosting (XGBoost), and Light Gradient Boosting Machine (LightGBM), based on sociodemographic, climate, landscape, and dengue surveillance epidemiological data (January 2000 to December 2021). The optimal tree‐based ML model with strong interpretability was created by comparing various ML models using the hyperparameter optimization technique. The feature importance ranking and the most significant dengue driver were found using the SHapley Additive explanation (SHAP) value.

**Results:**

Our study findings detected a nonlinear effect of climatic parameters on dengue at different thresholds such as mean (27°C), minimum (22°C), maximum temperatures (32°C), and relative humidity (82%). The optimal minimum and maximum temperatures, humidity, rainfall, and wind speed for dengue risk are 25−28°C, 32−34°C, 75%−85%, 10 mm, and 12 m/s, respectively. The LightGBM model accurately forecasts DF and agricultural land, population density, and minimum temperature significantly affecting the dengue outbreak in Bangladesh.

**Conclusion:**

Our proposed ML model functions as an early warning system, improving comprehension of the factors that precipitate dengue outbreaks and providing a framework for sophisticated analytical techniques in public health.

## 1 Introduction

Dengue is a globally perilous vector‐borne illness induced by the dengue virus (DENV) and disseminated among humans by female *Aedes* (*Ae.*) *aegypti* or *Ae. albopictus* mosquitoes [1, 2]. The disease is currently prevalent in more than 100 countries spanning the WHO's Regions of Africa, Americas, Southeast Asia, Eastern Mediterranean, and Western Pacific [3, 4]. Asia accounts for over 70% of the global disease burden, with the Americas, Southeast Asia, and Western Pacific regions being the most impacted [2]. The year 2023 witnessed the most dengue cases ever recorded. The WHO Region of the Americas reported 2300 deaths and 4.5 million cases. Bangladesh (321,000), Malaysia (111,400), Thailand (150,000), and Vietnam (369,000) reported a disproportionately high number of dengue cases in Asia [2, 3]. The rapid increase in DENV's global spread in recent decades has been attributed to several factors, including inadequate vector control efforts, urbanization, population growth, and climate change [5, 6]. In Bangladesh, dengue fever (DF) is endemic and continues to pose a serious threat to public health due to its recent increase in frequency and enlarged geographic range. The country reports an estimated 2.4 million cases of DF annually; 40 million people, or 24% of the population, have been infected with dengue at some point in their lives [7, 8, 9, 10, 11]. Following the first DF epidemics in Asia in 1779–1780, Bangladesh recorded the first dengue outbreak in 1964, also called “Dacca Fever.” The inaugural DF epidemic transpired in mid‐2000, with 5551 reported infections and a case fatality rate of 1.7%, resulting in 93 deaths [12]. There were between 3000 and 7000 hospitalized cases of dengue between 2002 and 2018 [12]. In 2019, 164 deaths and 354 cases were reported in the country [13, 14]. This DF outbreak, in terms of both cases and fatalities, was the bloodiest and most widespread in Bangladeshi history. Numerous studies indicate that sociodemographic factors can impact DENV transmission as profoundly as climatic factors [7, 15]. Some studies have shown that landscape factors may influence DF [15, 16, 17]. Countries like Bangladesh, which are frequently the most afflicted by DENV, often lack the finances and cutting‐edge technologies required to combat the disease. Developing a reliable and accurate forecasting model and improving the predictability of DF in Bangladesh have proven complex tasks [7, 15]. Both climate variables (temperature, rainfall, humidity, etc.) and non‐climatic factors such as socio‐ecological factors, for example, demographic, socioeconomic, behavioral, and social factors, and landscape variables tend to increase the disease risk in the country [7, 11, 18, 19].

Forecasting is essential in early warning systems (EWS) for disease epidemics because of the absence of effective dengue treatment alternatives and the restricted supply of vaccinations [18]. The WHO emphasized the necessity of creating predictive algorithms to evaluate the dengue risk in its worldwide strategy for dengue prevention and control [19, 20]. A variety of models have been employed to forecast disease outbreaks. A spatial‐temporal analysis framework was developed by combining random forest (RF) regression with a multi‐objective optimization algorithm to predict daily case numbers and mortality rates in Asia [20]. The predictive performance of support vector regression models and stacking‐ensemble learning techniques surpassed those of comparative models in Brazil [21]. The Prophet model was deemed to possess dependable predictive capability in South Korea [22]. Furthermore, with the ongoing advancement of computer science and software technology, artificial intelligence (AI) is increasingly being implemented in medical systems for disease detection and clinical diagnosis [23]. Machine learning (ML), particularly deep learning, is considered an essential component of AI [24] and has been extensively utilized in dengue prediction [23]. Deep learning techniques have garnered significant attention recently due to their exceptional nonlinear approximation abilities in time series analysis and their notable versatility. Nonetheless, it is essential to recognize that the interpretability of deep learning models is limited by their black‐box characteristics, and associated research has struggled to thoroughly examine the relationships between influencing factors and diseases. The strong estimates and improved prediction accuracy of ML models have been generally acknowledged, yet these models have not been actively employed to forecast dengue outbreaks in Bangladesh. Nonetheless, there is still a need to thoroughly evaluate tree‐based ML models for accurate DENV risk prediction in Bangladesh based on climate, sociodemographic, and landscape characteristics. This study employed three tree‐based models: RF, eXtreme Gradient Boosting (XGBoost), and Light Gradient Boosting Machine (LightGBM) to evaluate the predictive accuracy of socioeconomic factors, landscape patterns, and climatic influences on DENV outbreaks in Bangladesh from 2000 to 2021. We also estimated the models' ability to predict the temporal pattern of the DENV outbreaks in Bangladesh in 2018 and 2019. We developed a prediction framework by identifying the most significant DENV driver utilizing the SHapley Additive explanation (SHAP) value and high‐performance ML models. This is the first attempt to comprehensively compare various tree‐based ML methods for DF incidence prediction in Bangladesh. Our proposed ML model functions as an EWS to enhance understanding of the factors that trigger dengue outbreaks and provides a framework for the effective implementation of advanced analytical techniques in public health.

## 2 Materials and Methods

### 2.1 Study Location

Bangladesh, situated in Southeast Asia, is positioned between 20°59′ N to 26°63′ N and 88°03′ E to 92°67′ E, encompassing both tropical and subtropical climates. The seasons of the country are generally classified as summer (March to June; hot and humid), monsoon (June to October; warm and wet), and winter (October to March; cold and dry). In summer, the maximum temperature can reach up to 40°C; in winter, most parts of the nation have average lows of 10°C. Rainfall amounts vary from 1500 to 3000 mm on average per year in the country.

### 2.2 Data Source

**Figure 1:**
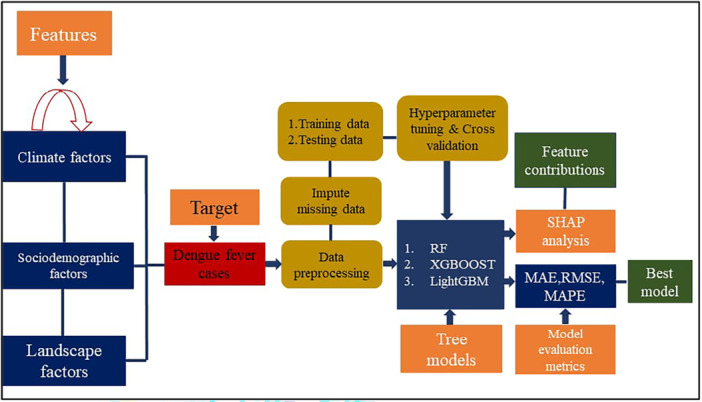
Conceptual framework of tree‐based ML model for predicting dengue cases. LightGBM, light gradient boosting; RF, random forest; XGBoost, eXtreme gradient boosting.

Our research data were from January 2000 to December 2021 and included dengue cases, climate, sociodemographics, and landscape factors. The predictive models were developed with a variety of variables (Figure 1), including dengue cases, climate (temperature, relative humidity, rainfall, surface pressure, and wind speed), sociodemographic factors, and landscape (forest area, agricultural land, arable land, and waste collection) characteristics (Table 1). The Directorate General of Health Services (DGHS), under the Ministry of Health and Family Welfare, Bangladesh, and the Institute of Epidemiology, Disease Control and Research (IEDCR), Bangladesh, officially released press statements to confirm the dengue‐related statistics. The two portals from which we gathered and consolidated all published data were https://old.dghs.gov.bd/index.php/bd/ and https://iedcr.gov.bd/surveillances/. This study's climate factors are obtained from the National Aeronautics and Space Administration (NASA) monthly estimated data set. This study retrieved sociodemographic and landscape factors from the World Bank and Bangladesh Waste Database (BWD).

**Table 1:** Input features of interpretable tree‐based ML models and data source.

Category	Code	Variables	Source	Temporal granularity
Climate	x1	Average temperature (°C)	NASA	Monthly
	x2	Minimum temperature (°C)	NASA	Monthly
	x3	Maximum temperature (°C)	NASA	Monthly
	x4	Relative humidity (%)	NASA	Monthly
	x5	Rainfall (mm)	NASA	Monthly
	x6	Surface pressure (kPa)	NASA	Monthly
	x7	Wind speed at 50 m maximum (m/s)	NASA	Monthly
Sociodemographic	x8	Population density	World Bank	Yearly
	x9	Gross domestic product (billion US$)	World Bank	Yearly
	x10	Gross national income (K US$)	World Bank	Yearly
	x11	Poverty headcount ratio (% of population)	World Bank	Yearly
	x12	Adult literacy rate (% of population)	World Bank	Yearly
	x13	Total unemployment (% of total labor force)	World Bank	Yearly
	x14	Access to electricity (% of population)	World Bank	Yearly
	x15	Safe sanitation service (% of population)	World Bank	Yearly
	x16	Population growth	World Bank	Yearly
	x17	Air transport, passengers carried	World Bank	Yearly
	x18	Current health expenditure	World Bank	Yearly
Landscape	x19	Forest area (% of total land area)	World Bank	Yearly
	x20	Agricultural land (% of total land area)	World Bank	Yearly
	x21	Arable land (% of the total land area)	World Bank	Yearly
	x22	Waste collection (tons)	BWD 2014	Yearly

Abbreviations: BWD, Bangladesh waste database; NASA, National Aeronautics and Space Administration.

### 2.3 Modeling Approach

### 2.4 Data Preprocessing

#### 2.4.1 Splitting Data Set

This study exclusively examined dengue outbreaks (≥ 688 cases). After imputing missing data using a KNN approach, the final data set was spilled into training and testing sets. The model was trained using data from 2000 to 2018, while data from 2019 to 2021 was designated for testing. Data analysis was performed using R software, version 4.2.2.

#### 2.4.2 Cross‐Validation

We chose the more computationally demanding but reliable k‐fold cross‐validation strategy to prevent overfitting or underfitting issues. A 10‐fold cross‐validation technique was used for each model's training data set.

#### 2.4.3 Hyperparameter Optimization

We pursued the optimal parameter combinations for three ML algorithms through hyperparameter optimization techniques. We utilized 10‐fold cross‐validation and employed the mean absolute percentage error (MAPE) as the objective function for optimization within the specified parameter space [25, 26].

### 2.5 ML Models

#### 2.5.1 RF

RF, a widely utilized and efficient supervised learning technique for regression and classification problems, is founded on bagging integration [27]. The RF model is articulated as Equation (1), with *P* denoting the quantity of decision trees.

(1) $$Y=\frac{1}{P}\sum_{i=1}^{P}\mathrm{Fi}(x)$$

#### 2.5.2 XGBOOST

In 2016, Chen Tianqi and Carlos Guestrin introduced the XGBoost algorithm, which integrates many weak learners to enhance the overall training and prediction's accuracy and robustness. The XGBoost algorithm is encapsulated by Equation (2), where l represents the loss function, fj signifies a weak learner, and Ω indicates the regularization term.

(2) $$L^{j}=\sum_{i=1}^{n}l\left\{\left(y_{i}{+y}_{i}^{j+1}+\text{fj}\left(\text{xj}\right)\right)\right\}+\Omega (\text{fj})$$

#### 2.5.3 LightGBM

The LightGBM is a histogram‐based decision tree model that uses two cutting‐edge methods to speed up computation and increase performance: exclusive feature aggregation and gradient‐based unilateral sampling. To ensure the precision of the information gain calculation, the initial strategy randomly excludes cases with minimal gradients while retaining instances with substantial gradients.

#### 2.5.4 SHAP Analysis

This study employs SHAP analysis which is comparatively simple to read and has quick implementations linked to numerous well‐known RF, XGBoost, and LightGBM ML models. The explanation model calculates the mean predicted value and the total contributions from each input feature to assess an ML system's predictions. Mathematically, the explanation model can be stated as:

(3) $$y=\overset{®}{y}+\sum_{i}\varphi$$

### 2.6 Model Evaluation Measurement

Our study employed three accuracy metrics to evaluate model performance: mean absolute error (MAE), MAPE, and root mean square error (RMSE) as detailed below:

(4) $$\mathrm{MAE}=\frac{1}{n}\sum_{i=0}^{n}\left|{\hat{y}}_{i}-y_{i}\right|$$

(5) $$\mathrm{RMSE}=\sqrt{\frac{1}{n}{\sum_{1}^{n}({\hat{y}}_{i}-y_{i})}^{2}}$$

(6) $$\mathrm{MAPE}=\frac{1}{n}\sum_{1}^{n}|\frac{{\hat{y}}_{i}-y_{i}}{y_{i}}|⨉100\%$$

where $y_{i}\;$denotes the observed values, ${\hat{y}}_{i}\;$represents prediction, and *n* signifies the number of data points.

## 3 Results

**Figure 2:**
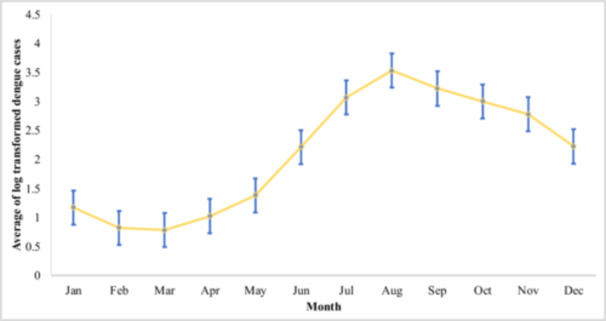
Monthly average dengue cases in Bangladesh from January 2000 to December 2021. The shaded region denotes the 95% confidence interval.

Dengue predominantly transpires from July to November annually between January 2000 and December 2021 (Figure 2). The mean values of daily temperatures were 25.5°C (mean), 18.4°C (minimum), and 33.2°C (maximum), while the averages for relative humidity, rainfall, surface pressure, and wind speed were 75.9%, 6.5 mm, 100.7 kPa, and 10.1 m/s, respectively (Table 2).

**Table 2:** Descriptive statistics of monthly climate factors and dengue cases in Bangladesh during January 2000 to December 2021.

Factors	Mean ± SD	Minimum	Median	Maximum
Dengue cases	688 ± 3655	0	31.0	52636
Average temperature (°C)	25.5 ± 4.1	15.5	27.5	31.9
Minimum temperature (°C)	18.4 ± 6.4	3.4	20.3	26.3
Maximum temperature (°C)	33.2 ± 3.8	24.6	32.9	42.5
Relative humidity (%)	75.9 ± 13.3	45.2	79.7	91.7
Rainfall (mm)	6.5 ± 9.9	0	4.8	131.3
Surface pressure (kPa)	100.7 ± 0.5	99.8	100.7	101.6
Wind speed (m/s)	10.1 ± 2.5	5.3	9.8	20.6

Abbreviation: SD, standard deviation.

### 3.1 Climate, Sociodemographic, and Landscape Characteristics

**Figure 3:**
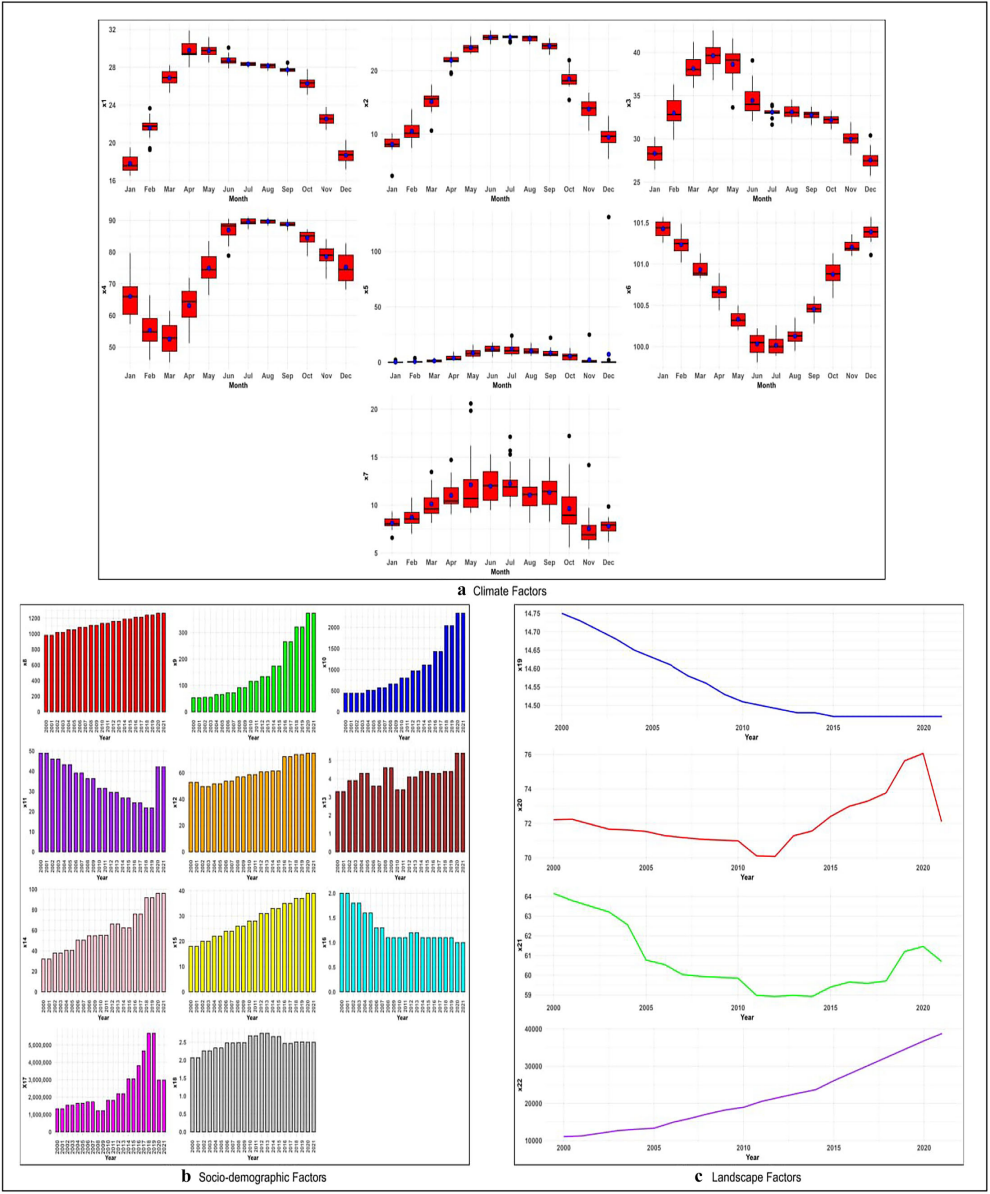
(a) Boxplot of monthly mean distribution of climate factors during January 2000 to December 2021. X1: average temperature (°C); X2: minimum temperature (°C); X3: maximum temperature (°C); X4: relative humidity (%); X5: precipitation (mm); X6: atmospheric pressure (kPa); X7: maximum wind speed at 50 m (m/s). Potential outliers were presented in black circles, and the mean value was in blue circles. (b) Histogram recorded sociodemographic factors during January 2000 to December 2021 in Bangladesh. X8: population density; X9: gross domestic product (billion US$); X10: gross national income (K US$); X11: poverty head‐count ratio (% of population); x12: adult literacy rate (% of population); X13: total unemployment (% of total labor force); X14: access to electricity (% of population); X15: safe sanitation service (% of population); X16: population growth; X17: air transport, passengers carried; X18: current health expenditure. (c) Time series plot of landscape factors in Bangladesh during January 2000 to December 2021. X19: forest area (% of total land area); X20: agricultural land; X21: arable land; X22: waste collection.

The monthly average temperature in the country rose after January and persisted in its ascent until June/July (Figure 3a). The monthly minimum temperature increased between April to September (Figure 3a). Rainfall escalated from April to October (Figure 3a). Rainfall peaks typically between May and August, while minimal rainfall is observed in January, February, and December.

There were considerable changes in climate (Figure 3a), sociodemographic (Figure 3b), and landscape factors (Figure 3c) in the country during 2000−2021 (Figure 3). Forest area and land were decreased in the country during the study period (Figure 3c). The waste collection was frequent in the country (Figure 3c).

### 3.2 Correlation Between Dengue and Climate, Sociodemographic, and Landscape Variables

Pearson's correlation analysis showed that all four climate factors (relative humidity, rainfall, minimum temperature) except surface pressure were positively associated with DF incidence (Supporting Information S1: Figure S1). All eight sociodemographic factors except poverty head‐count ratio and adult literacy rate positively affected dengue incidence (Supporting Information S1: Figure S2). Moreover, two significant landscape factors, agricultural land, and waste collection, positively affect dengue incidence (Supporting Information S1: Figure S3). The monthly average, minimum and maximum temperatures, relative humidity, rainfall, and surface pressure exhibited a positive linear relationship at lower levels until a specified threshold was attained; conversely, rainfall demonstrated a linear effect beyond a threshold (Supporting Information S1: Figure S4). A threshold of 27°C, 22°C, and 32°C was established for monthly average, minimum, and maximum temperatures, respectively; 82% for relative humidity; 7 mm for rainfall; 100 kPa for surface pressure; and 7 m/s for wind speed concerning DF risk (Supporting Information S1: Figure S4). We identified a linear correlation between temperature and relative humidity below the threshold, and rainfall above the threshold.

### 3.3 Performance Measurements of All Models

**Figure 4:**
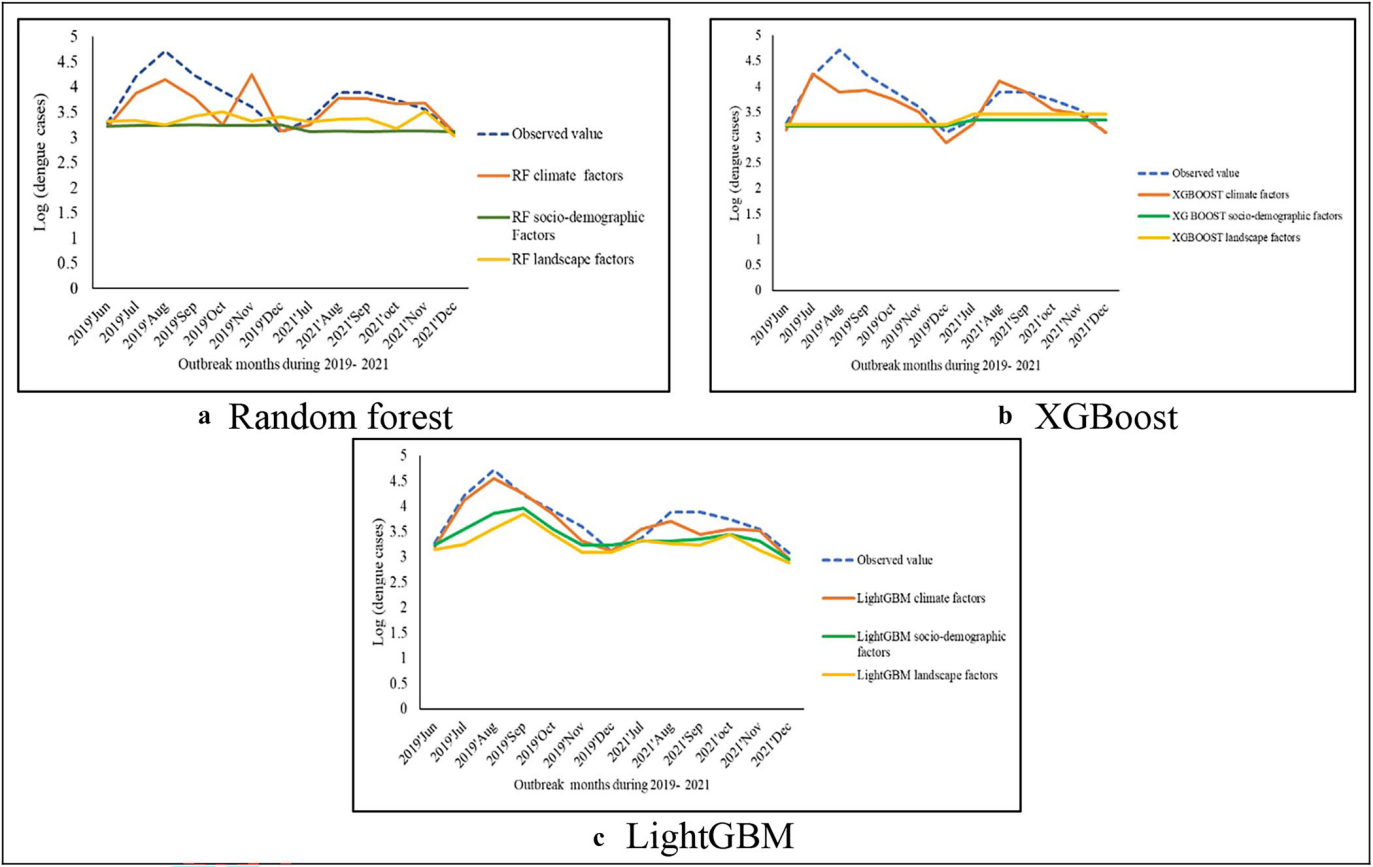
Prediction accuracy of climate, sociodemographic, and landscape factors of dengue cases during 2019−2021 using (a) random forest model, (b) XGBoost model, and (c) LightGBM modeling techniques.

Table 3 presents the specifics of the model evaluation criteria. Among the three principal ML models, the predictive accuracy is ordered as follows: LightGBM, XGBoost, and RF (Figure 4). The LightGBM model precisely predicts the DF in Bangladesh.

**Table 3:** Performance measurement of tree‐based ML models.

Tree models	Category	Data set	MAE	RMSE	MAPE
RF	Climate	Test data set	0.65	0.71	0.162
		Training data set	0.42	0.67	0.154
	Sociodemographic	Test data set	0.85	0.79	0.166
		Training data set	0.48	0.56	0.136
	Landscape	Test data set	0.87	0.78	0.147
		Training data set	0.47	0.65	0.125
XGBOOST	Climate	Test data set	0.51	0.62	0.131
		Training data set	0.42	0.58	0.121
	Sociodemographic	Test data set	0.53	0.68	0.169
		Training data set	0.52	0.53	0.132
	Landscape	Test data set	0.54	0.67	0.161
		Training data set	0.41	0.54	0.135
LightGBM	Climate	Test data set	0.28	0.36	0.091
		Training data set	0.24	0.32	0.053
	Sociodemographic	Test data set	0.46	0.53	0.108
		Training data set	0.41	0.42	0.082
	Landscape	Test data set	0.47	0.57	0.107
		Training data set	0.34	0.42	0.094

Abbreviations: LightGBM, light gradient boosting machine; MAE, mean absolute error; MAPE, mean absolute percentage error; RF, random forest; RMSE, root mean square error; XGBoost, eXtreme gradient boosting.

Furthermore, an analysis of model performance across the three data set types revealed that the LightGBM‐climate, LightGBM‐sociodemographic, and LightGBM‐landscape models exhibited lower MAE, RMSE, and MAPE values compared to the other models (Table 3).

### 3.4 Feature Importance

**Figure 5:**
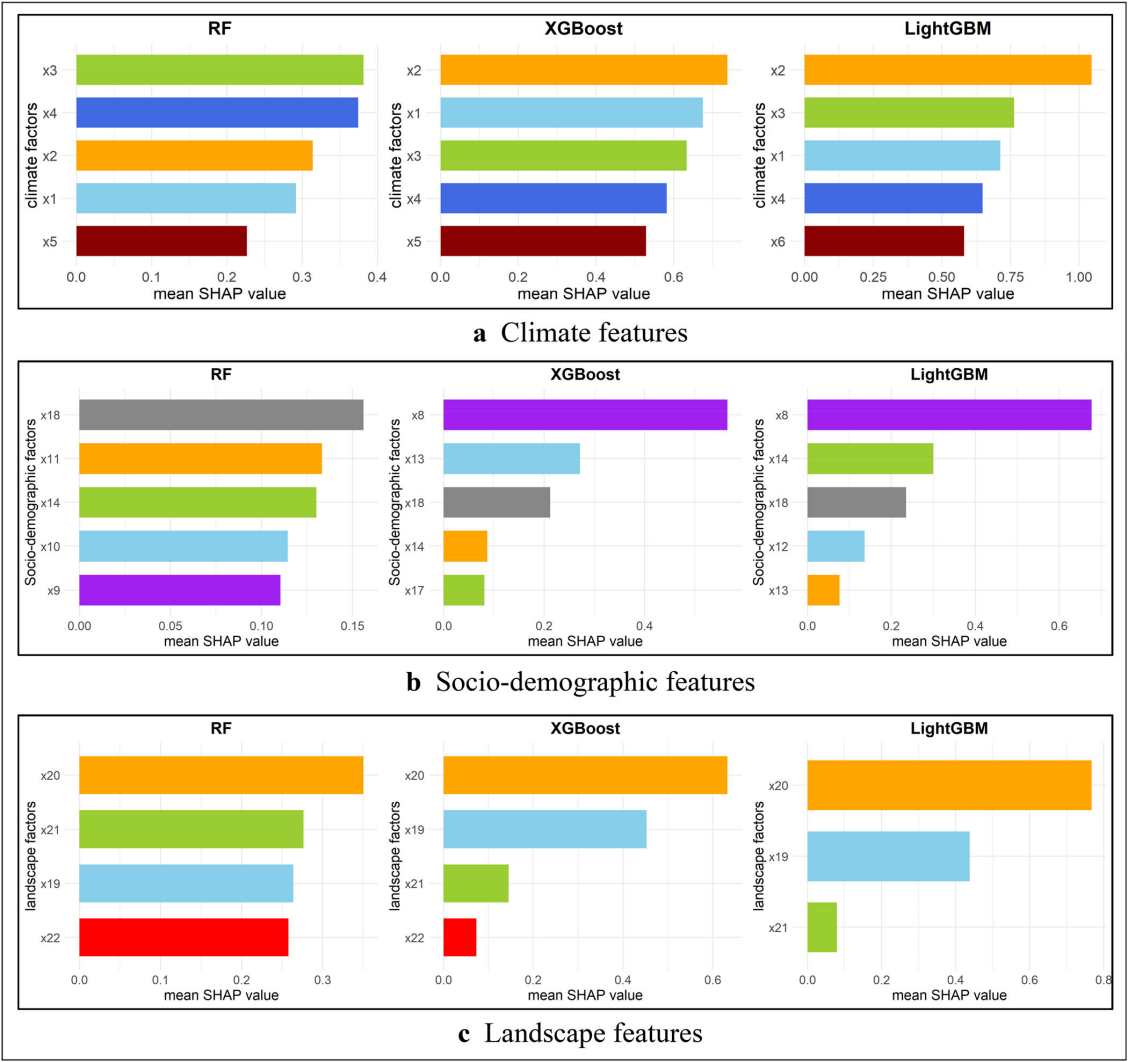
(a) Analysis of feature importance utilizing SHAP values in relation to climatic factors. X1: average temperature (°C); X2: minimum temperature (°C); X3: maximum temperature (°C); X4: relative humidity (%); X5: rainfall (mm); X7: wind speed at 50 m maximum (m/s). (b) Sociodemographic factors. X8: population density; X12: adult literacy rate (% of population); X13: total unemployment (% of total labor force); X15: safe sanitation service (% of population); X16: population growth; X18: current health expenditure. (c) Landscape factors. X19: forest area; X20: agricultural land; X21: arable land; X22: waste collection.

Employing the SHAP methodology, we extracted the feature SHAP values for the three primary ML techniques and subsequently evaluated feature significance based on the weights derived from the optimal ensemble method. The mean SHAP absolute value indicates the average impact on the magnitude of the model output. Figure 5a illustrates the most significant climatic attributes influencing the results of the RF, XGBoost, and LightGBM models. Figure 5b illustrates the most significant sociodemographic characteristics influencing the outcomes of the RF, XGBoost, and LightGBM models. Figure 5c illustrates the most significant landscape features influencing the outcomes of the RF, XGBoost, and LightGBM models.

**Figure 6:**
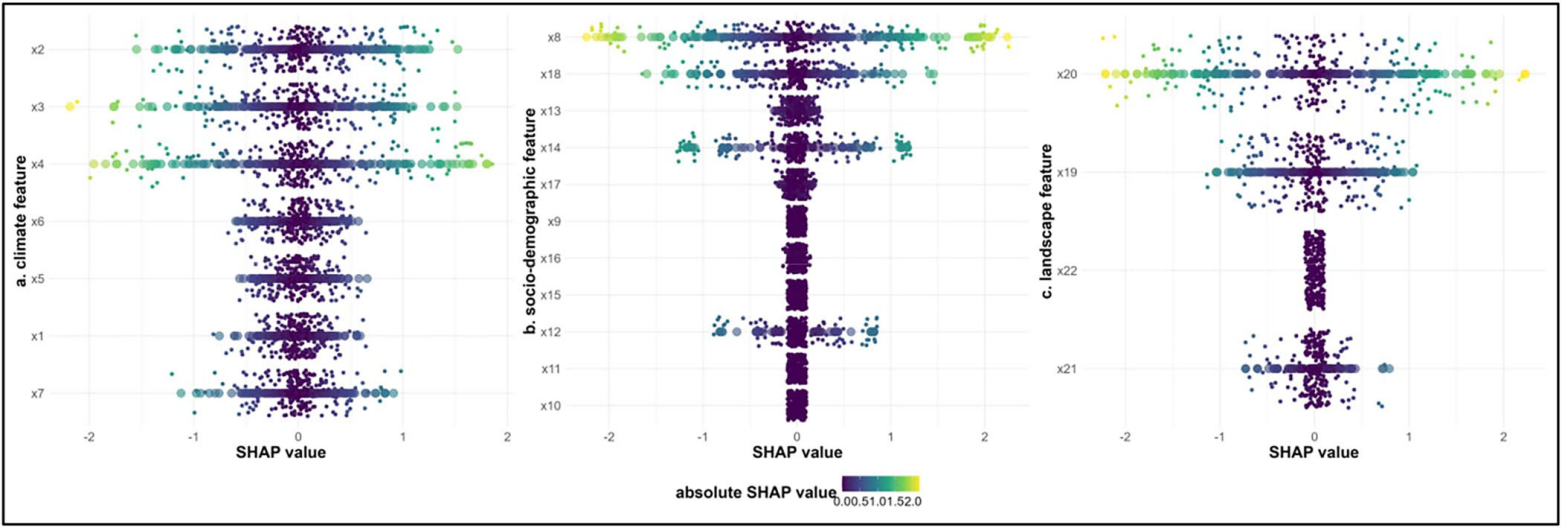
(a) Important SHAP predicted climate features of LightGBM model in Bangladesh during January 2000 to December 2021. X1: average temperature (°C); X2: minimum temperature (°C); X3: maximum temperature (°C); X4: relative humidity (%); X5: rainfall (mm); X 6: surface pressure (kPa); X7: wind speed at 50 m maximum (m/s). (b) Important SHAP predicted sociodemographic features of LightGBM model in Bangladesh during January 2000 to December 2021. X8: population density; X9: gross domestic product (billion US$); X10: gross national income (K US$); X11: poverty head‐count ratio (% of population); x12: adult literacy rate (% of population); X13: total unemployment (% of total labor force); X14: access to electricity (% of population); X15: safe sanitation service (% of population); X16: population growth; X17: air transport, passengers carried; X18: current health expenditure. (c) Important SHAP predicted landscape features of LightGBM model in Bangladesh during January 2000 to December 2021. X20: agricultural land; X21: arable Land; X22: waste collection (tons).

The lowest temperature had the highest impact on dengue prediction (Supporting Information S1: Table S8), with a mean SHAP value of 0.938 (Figure 6a). Population density was the most significant feature in the sociodemographic category, with a mean SHAP value of 0.658 (Figure 6b). Agricultural land was found to be the most significant landscape feature in dengue prediction, with a mean SHAP value of 0.730 (Figure 6c).

**Figure 7:**
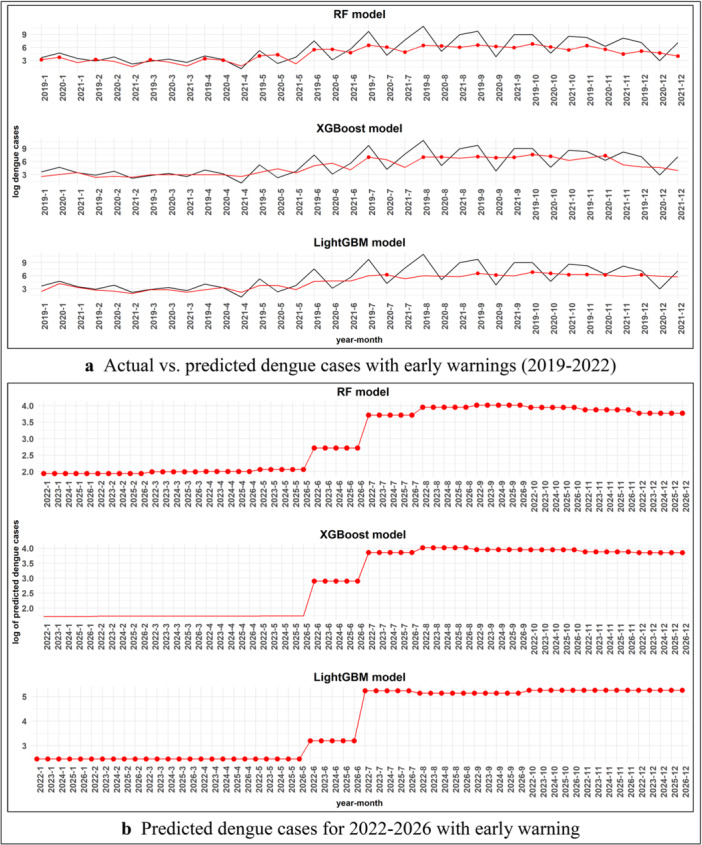
Forecasting and early warning system for dengue cases: (a) actual versus predicted dengue cases with early warnings (2019−2022), (b**)** predicted dengue cases for 2022−2026 with early warnings.

The comparative analysis of RF, XGBoost, and LightGBM in predicting dengue cases reveals notable differences in performance and sensitivity (Supporting Information S1: Tables S1−S7, Figure 7 and Supporting Information S1: Figure S5). These differences were influenced by various climate, sociodemographic, and landscape factors, which played a critical role in shaping the models' ability to predict outbreaks accurately and issue early warnings. Our proposed model exhibited robust performance from June to October, coinciding with the peak dengue season, especially in August and October 2019. The model's predictions were highly influenced by climate factors including mean temperature and rainfall, leading to frequent warnings during high‐risk months. However, RF showed lower sensitivity in 2020 and 2021, failing to issue warnings during February and March 2020 when predictions were relatively low (Supporting Information S1: Table S1 and Figure 7a). This suggested RF's reduced ability to predict outbreaks during periods of lower‐case numbers, highlighting its dependence on climate variables.

XGBoost model demonstrated higher sensitivity, especially in detecting rising dengue cases (Supporting Information S1: Table S3, Figure 7a). In August 2020, it predicted 1174.41 cases, marking a significant increase. The model was more responsive to changes in relative humidity and wind speed, which helped it detect emerging outbreaks later in the year, issuing a warning in September 2020. This illustrates XGBoost's ability to capture trends and predict outbreaks more effectively than RF, especially when influenced by climate and landscape factors. LightGBM took a more conservative approach, issuing fewer warnings overall (Supporting Information S1: Table S5, Figure 7a). Despite fewer warnings, it successfully identified major outbreaks in September and October 2020, showing strong reliance on sociodemographic factors, such as GDP (x9) and population density (x8), which likely contributed to its more selective approach. While it was less proactive in early detection, LightGBM's ability to focus on larger, more densely populated areas effectively predicted high‐risk months and minimized false positives.

The long‐term predictions for 2022−2026 (Supporting Information S1: Table S6, Figure 7b), further emphasize the differences between the models: RF and XGBoost continued to issue frequent warnings, while LightGBM provided fewer but more targeted alerts, focusing on high‐risk months. This highlights how the models differ in approach, with RF and XGBoost being more proactive and LightGBM adopting a more selective prediction strategy influenced by climate, sociodemographic, and landscape factors.

## 4 Discussion

Since 2010, every seasonal epidemic has seen an increase in dengue incidence in Bangladesh, except for 2014 [8, 12, 28]. In 2019, the dengue outbreak peaked in the country [29]. This study showed that landscape, sociodemographic, and climatic factors influence Bangladesh's dengue incidence. This is the first study of its kind in the nation to show that dengue expansions in Bangladesh during the previous 22 years can be explained by integrating variability in the country's climate, sociodemographics, and geography before dengue season. This indicates that dengue EWS could be established for this country utilizing our proposed model. Our study findings revealed a positive correlation between minimum temperatures from January to March and dengue cases; however, from April to June, as the dengue season approached, a negative correlation was observed. There was a positive correlation between maximum temperatures in December and March and dengue; however, there was a negative correlation from April through June. The complicated relationship between the dengue vector's population dynamics and the environment, including the seasonal shift from winter to summer and the rising temperatures that accompany it, could be the cause [28, 30, 31, 32, 33, 34].

A study asserted that temperatures ranging from 21°C to 34°C are ideal for the proliferation of *Ae. aegypti* populations. Our study indicates an optimal daily minimum and maximum range of 22°–25°C and 32°–34°C, respectively, are the risk factors of DF. Rainfall exerts both advantageous and detrimental influences on the proliferation of mosquito populations [35, 36]. Rainfall can create stagnant water conducive to mosquito reproduction. Our study has revealed an inverse correlation between rainfall and dengue incidence in January. This may be attributed to the low population of adult mosquitoes during this period, resulting in rainfall having a more detrimental effect by washing away mosquito eggs than a beneficial effect by providing habitats necessary for adult mosquitoes [8, 35, 36, 37]. Recently conducted studies in Hong Kong and Taiwan have demonstrated a comparable trend of a negative correlation between pre‐dengue‐season rainfall and dengue cases [38, 39]. The study established a threshold of 82% for the impact of relative humidity, consistent with the findings of a corresponding study conducted in China [40, 41, 42]. Relative humidity positively influences the incidence of DF when below the threshold. Optimal humidity has been reported to enhance mosquitoes' oviposition, egg‐hatching, and feeding behavior [42].

The SHAP analysis and results from the LightGBM model offer significant insights into the determinants of dengue outbreaks. These results highlight the critical role of climatic, sociodemographic, and landscape features in predicting dengue risk. According to the suggested LightGBM ML model, climate and landscape factors are more significant in forecasting dengue incidence in the country than sociodemographic characteristics. This finding aligns with a study conducted in the United States, which revealed that landscape variables were more influential than sociodemographic factors in predicting dengue incidence [15]. Agricultural land positively influences dengue incidence; analogous results are observed in Thailand, where heightened rice production resulting from forest conversion constitutes a significant land‐use alteration impacting dengue forecasting [43, 44, 45]. This study showed that waste collection also positively affects dengue incidence in Bangladesh. This finding is similar to a recent study in the Maldives [46]. Our study demonstrated that population density was the most influential sociodemographic factor. Some previous studies had similar findings where population density positively affected the dengue incidence. High population density increases human‐mosquito interactions and creates favorable environments for mosquito breeding in densely populated urban areas. This finding highlights the need for targeted vector control strategies in urban areas, alongside improved sanitation infrastructure to reduce stagnant water where mosquitoes breed. Other sociodemographic factors, such as access to electricity and adult literacy rates, also indirectly influence dengue outcomes. Access to electricity can enhance healthcare infrastructure, aiding in better outbreak management, while higher literacy rates help improve public awareness and engagement in preventive measures. Agricultural areas often rely on irrigation systems, which can create standing water, thus serving as ideal breeding grounds for mosquitoes. The SHAP analysis suggests that sustainable agricultural practices, such as water‐efficient irrigation and proper drainage systems, can help mitigate mosquito breeding. Although forest areas and arable land had less impact on dengue transmission, their management is still relevant in reducing mosquito proliferation in these regions. The combination of these findings demonstrates the importance of integrated strategies that consider climate, sociodemographic, and landscape factors in predicting and managing dengue outbreaks.

Out of all the ML models employed, LightGBM was determined to be suitable for detecting subtle interplays between various variables and yielded excellent precision in this study. Therefore, the LightGBM ML model can reliably estimate and predict dengue incidence in Bangladesh across various climate, sociodemographic, and landscape variables. Public health professionals can control infectious disease outbreaks by utilizing the integrated evidence that our proposed ML model provides. Our results emphasize the need for a multifaceted approach to dengue control. Effective strategies should involve monitoring and managing temperature trends, reducing population density in high‐risk areas, and implementing water‐efficient agricultural practices. Furthermore, strengthening urban sanitation systems and promoting public health campaigns focused on mosquito breeding prevention can significantly reduce dengue transmission. Future research should explore the interactions between these factors in greater detail and refine prediction models to improve outbreak mitigation strategies. By synthesizing these findings, policymakers and public health officials can formulate more precise and effective strategies to avert dengue outbreaks in at‐risk areas. The integration of SHAP analysis with ML models, as demonstrated in studies such as Alabdullah et al. for concrete prediction, highlights the value of these advanced techniques in providing a more comprehensive understanding of complex systems and improving decision‐making processes in public health [47].

## 5 Conclusion

Our study utilized an interpretable tree‐based LightGBM ML model to forecast dengue outbreaks in Bangladesh, ensuring optimal predictive performance. Our study findings illustrated that minimum temperature, population density, and agriculture significantly impact dengue outbreaks. Our proposed ML model can serve as a public health EWS to prevent dengue outbreaks in Bangladesh and similar settings elsewhere. Essentially, this study advances knowledge about the risk factors of dengue outbreaks and promotes multidisciplinary cooperation and the useful application of cutting‐edge analytical techniques in public health.

## Author Contributions

**Md. Siddikur Rahman:** conceptualization, investigation, writing – original draft, funding acquisition, writing – review and editing, validation, methodology, visualization, project administration, formal analysis, software, data curation, supervision, resources. **Miftahuzzannat Amrin:** visualization, data curation, writing – review and editing. **Md. Abu Bokkor Shiddik:** data curation, visualization, writing – review and editing.

## Ethics Statement

The authors have nothing to report.

## Conflicts of Interest

The authors declare no conflicts of interest.

## Transparency Statement

The lead author, Md. Siddikur Rahman affirms that this manuscript is an honest, accurate, and transparent account of the study being reported, that no important aspects of the study have been omitted and that any discrepancies from the study as planned (and, if relevant, registered) have been explained.

## Supporting information

Supplements.

## Data Availability

All the raw data were collected from the public database (https://old.dghs.gov.bd/index.php/bd/home/5200-daily-dengue-status-report). The necessary data and source codes are also available at https://github.com/siddikur2022/dengue_outbreaks_Bangladesh.
