# Supplementary material for: Dengue Early Warning System and Outbreak Prediction Tool in Bangladesh Using Interpretable Tree‐Based Machine Learning Model
Source: Health Sci Rep. 2025 May 9;8(5):e70726. doi: 10.1002/hsr2.70726 (PMC12063067; doi:10.1002/hsr2.70726)
Supplement: Supplementary file 1 — Supplements. [file HSR2-8-e70726-s001.docx]

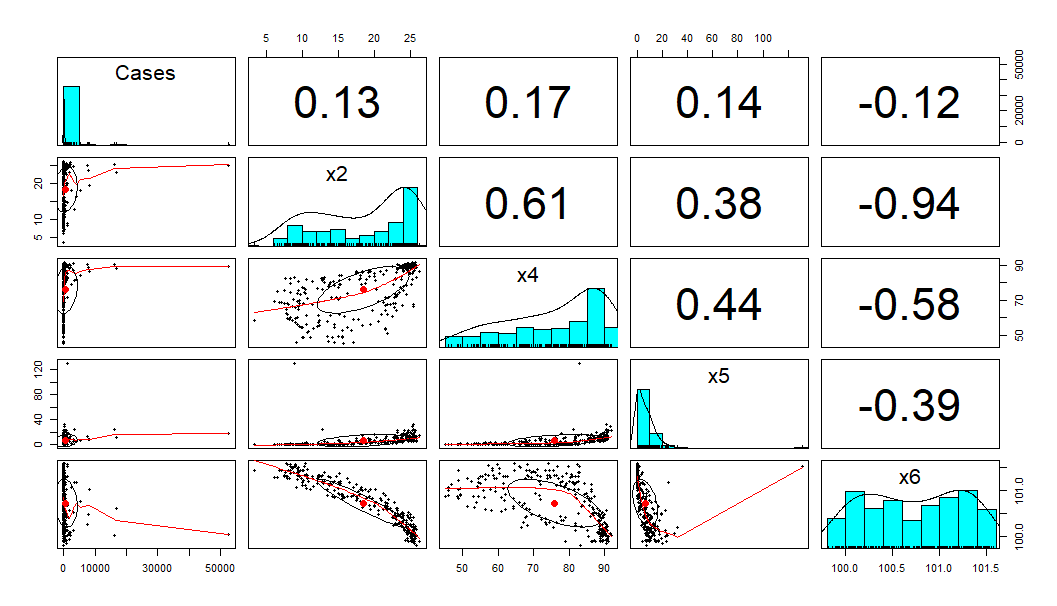


**Fig S1.** Pearson correlation between climate factors and dengue cases from January 2000 to December 2021. X2: Minimum temperature (°C); X4: Relative humidity (%); X5: Rainfall (mm); X6: Surface Pressure (kPa)


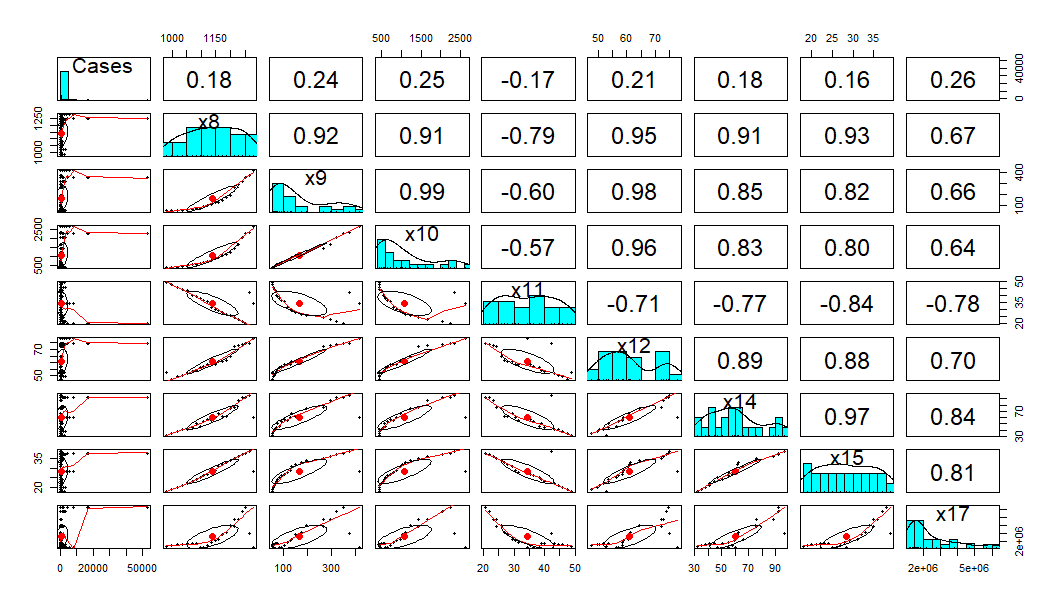


**Fig S2.** Pearson correlation between socio-demographic factors and dengue cases from January 2000 to December 2021. X8: Population density; X9: Gross Domestic Product (Billion US$); X10: Gross National Income (K US$); X11: Poverty head-count ratio (% of population); X12: Adult literacy rate (% of population); X14: Access to electricity (% of population); X15: Safe sanitation service (% of population); X17: Air transport, passengers Carried.


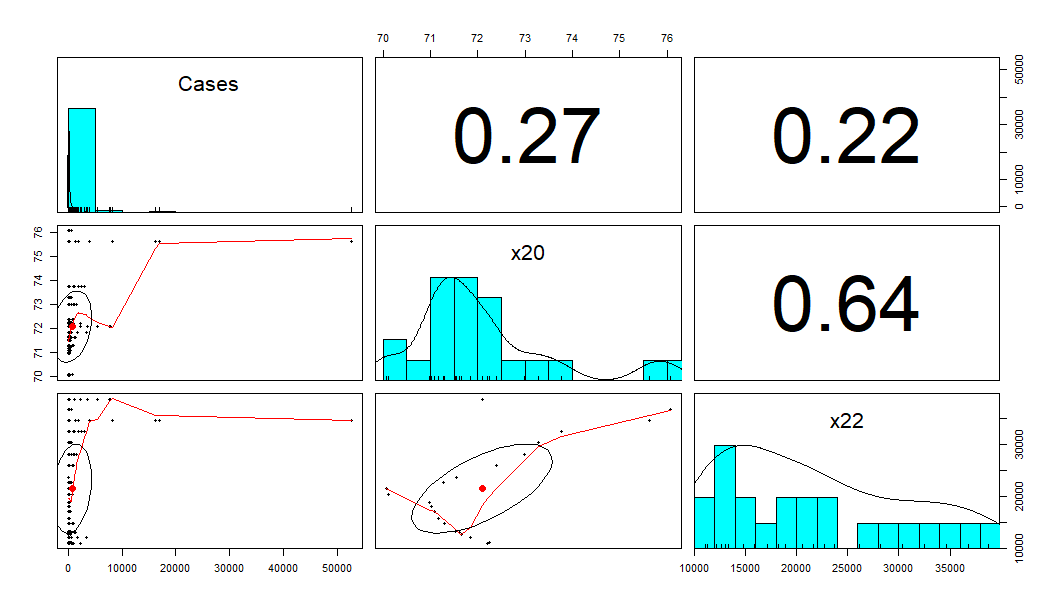


**Fig S3.** Pearson correlation between landscape factors and dengue cases from January 2000 to December 2021.X20: Agricultural land (% of total land area); X22: Waste collection (tons)


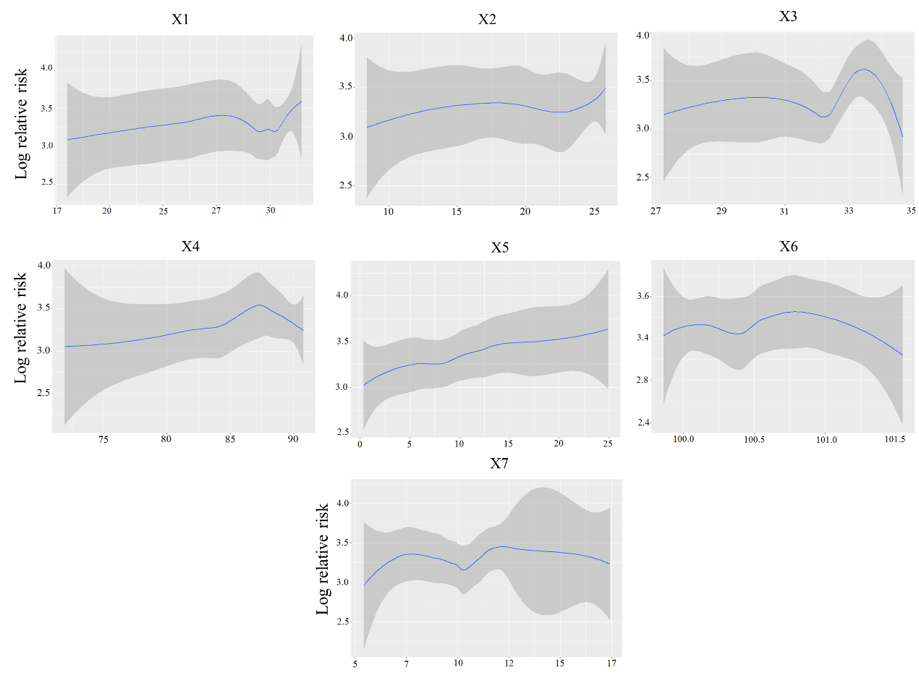


**Fig S4.** Smoothing plots of monthly climate factors and dengue cases during January 2000- December 2022. X1: Mean temperature (°C); X2: Minimum temperature (°C); X3: Maximum temperature (°C); X4: Relative humidity (%); X5: Rainfall (mm); X 6: Surface Pressure (kPa); X7: Wind Speed at 50 Meters Maximum (m/s)


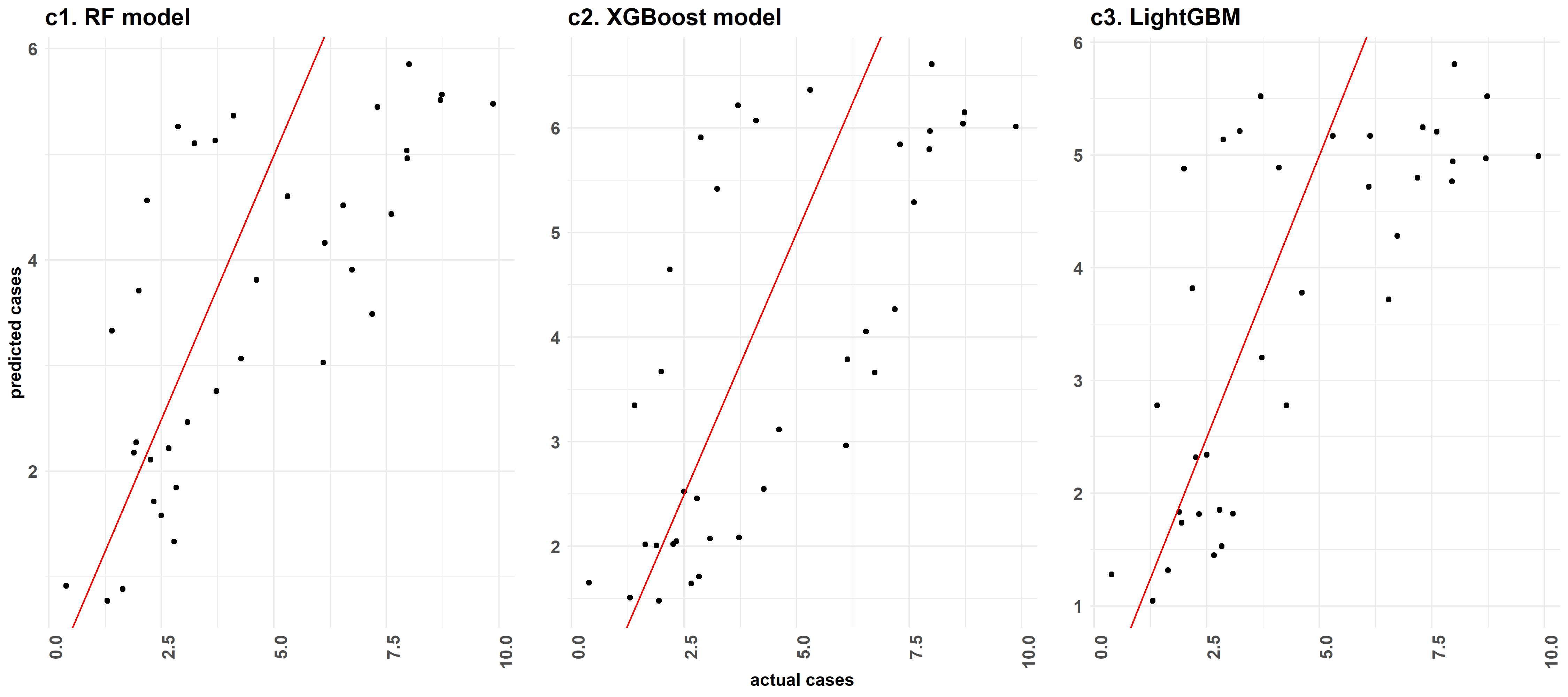


**Fig S5.** Comparison of actual vs. predicted dengue cases with c1. RF model, c2. XGBoost model snd c3. LightGBM model

**Table S1.** Monthly actual and predicted dengue cases with warning status by RF model (2019-2021)

| **Year** | **Month** | **Actual** | **Predicted** | **Warning** |
| --- | --- | --- | --- | --- |
| 2019 | January | 38 | 23.91 | Warning |
| 2019 | February | 18 | 25.38 | Warning |
| 2019 | March | 17 | 22.93 | Warning |
| 2019 | April | 58 | 30.89 | Warning |
| 2019 | May | 193 | 57.20 | Warning |
| 2019 | June | 1884 | 247.44 | Warning |
| 2019 | July | 16253 | 672.95 | Warning |
| 2019 | August | 52636 | 649.91 | Warning |
| 2019 | September | 16856 | 708.97 | Warning |
| 2019 | October | 8113 | 946.69 | Warning |
| 2019 | November | 4011 | 629.60 | Warning |
| 2019 | December | 1247 | 173.39 | Warning |
| 2020 | January | 111 | 41.82 | Warning |
| 2020 | February | 45 | 16.19 | No Warning |
| 2020 | March | 27 | 14.04 | No Warning |
| 2020 | April | 25 | 21.35 | Warning |
| 2020 | May | 10 | 74.87 | Warning |
| 2020 | June | 23 | 259.97 | Warning |
| 2020 | July | 68 | 445.89 | Warning |
| 2020 | August | 163 | 579.49 | Warning |
| 2020 | September | 47 | 523.36 | Warning |
| 2020 | October | 109 | 459.25 | Warning |
| 2020 | November | 546 | 269.82 | Warning |
| 2020 | December | 19 | 110.01 | Warning |
| 2021 | January | 32 | 12.18 | No Warning |
| 2021 | February | 9 | 4.87 | No Warning |
| 2021 | March | 13 | 5.59 | No Warning |
| 2021 | April | 3 | 5.77 | No Warning |
| 2021 | May | 43 | 9.32 | No Warning |
| 2021 | June | 272 | 121.63 | Warning |
| 2021 | July | 2286 | 133.99 | Warning |
| 2021 | August | 7698 | 416.74 | Warning |
| 2021 | September | 7841 | 387.07 | Warning |
| 2021 | October | 5458 | 228.03 | Warning |
| 2021 | November | 3567 | 87.78 | Warning |
| 2021 | December | 1207 | 55.15 | Warning |

**Table S2.** Predicted dengue cases with warning status by RF model (2022-2026)

| **Year** | **Month name** | **Predicted** | **Warning** |
| --- | --- | --- | --- |
| 2022 | January | 6.947417 | No Warning |
| 2023 | January | 6.947417 | No Warning |
| 2024 | January | 6.947417 | No Warning |
| 2025 | January | 6.947417 | No Warning |
| 2026 | January | 6.947417 | No Warning |
| 2022 | February | 6.935307 | No Warning |
| 2023 | February | 6.935307 | No Warning |
| 2024 | February | 6.935307 | No Warning |
| 2025 | February | 6.935307 | No Warning |
| 2026 | February | 6.935307 | No Warning |
| 2022 | March | 7.299123 | No Warning |
| 2023 | March | 7.299123 | No Warning |
| 2024 | March | 7.299123 | No Warning |
| 2025 | March | 7.299123 | No Warning |
| 2026 | March | 7.299123 | No Warning |
| 2022 | April | 7.402988 | No Warning |
| 2023 | April | 7.402988 | No Warning |
| 2024 | April | 7.402988 | No Warning |
| 2025 | April | 7.402988 | No Warning |
| 2026 | April | 7.402988 | No Warning |
| 2022 | May | 7.857949 | No Warning |
| 2023 | May | 7.857949 | No Warning |
| 2024 | May | 7.857949 | No Warning |
| 2025 | May | 7.857949 | No Warning |
| 2026 | May | 7.857949 | No Warning |
| 2022 | June | 15.154004 | No Warning |
| 2023 | June | 15.154004 | No Warning |
| 2024 | June | 15.154004 | No Warning |
| 2025 | June | 15.154004 | No Warning |
| 2026 | June | 15.154004 | No Warning |
| 2022 | July | 40.944920 | No Warning |
| 2023 | July | 40.944920 | No Warning |
| 2024 | July | 40.944920 | No Warning |
| 2025 | July | 40.944920 | No Warning |
| 2026 | July | 40.944920 | No Warning |
| 2022 | August | 52.083753 | Warning |
| 2023 | August | 52.083753 | Warning |
| 2024 | August | 52.083753 | Warning |
| 2025 | August | 52.083753 | Warning |
| 2026 | August | 52.083753 | Warning |
| 2022 | September | 55.442829 | Warning |
| 2023 | September | 55.442829 | Warning |
| 2024 | September | 55.442829 | Warning |
| 2025 | September | 55.442829 | Warning |
| 2026 | September | 55.442829 | Warning |
| 2022 | October | 51.684260 | Warning |
| 2023 | October | 51.684260 | Warning |
| 2024 | October | 51.684260 | Warning |
| 2025 | October | 51.684260 | Warning |
| 2026 | October | 51.684260 | Warning |
| 2022 | November | 48.030557 | No Warning |
| 2023 | November | 48.030557 | No Warning |
| 2024 | November | 48.030557 | No Warning |
| 2025 | November | 48.030557 | No Warning |
| 2026 | November | 48.030557 | No Warning |
| 2022 | December | 43.480597 | No Warning |
| 2023 | December | 43.480597 | No Warning |
| 2024 | December | 43.480597 | No Warning |
| 2025 | December | 43.480597 | No Warning |
| 2026 | December | 43.480597 | No Warning |

**Table S3.** Monthly actual and predicted dengue cases with warning status by XGBoost model (2019-2021)

| **Year** | **Month name** | **Actual** | **Predicted** | **Warning** |
| --- | --- | --- | --- | --- |
| 2019 | January | 38 | 13.07116 | No Warning |
| 2019 | February | 18 | 10.90476 | No Warning |
| 2019 | March | 17 | 19.24145 | No Warning |
| 2019 | April | 58 | 20.63056 | No Warning |
| 2019 | May | 193 | 33.68221 | No Warning |
| 2019 | June | 1884 | 155.81819 | No Warning |
| 2019 | July | 16253 | 1137.75369 | Warning |
| 2019 | August | 52636 | 1108.20127 | Warning |
| 2019 | September | 16856 | 1271.27444 | Warning |
| 2019 | October | 8113 | 2016.53842 | Warning |
| 2019 | November | 4011 | 934.93369 | No Warning |
| 2019 | December | 1247 | 119.04247 | No Warning |
| 2020 | January | 111 | 20.85964 | No Warning |
| 2020 | February | 45 | 14.03938 | No Warning |
| 2020 | March | 27 | 20.04218 | No Warning |
| 2020 | April | 25 | 19.50986 | No Warning |
| 2020 | May | 10 | 76.34365 | No Warning |
| 2020 | June | 23 | 282.13400 | No Warning |
| 2020 | July | 68 | 610.32307 | No Warning |
| 2020 | August | 163 | 1174.40761 | Warning |
| 2020 | September | 47 | 998.81987 | Warning |
| 2020 | October | 109 | 1358.57079 | Warning |
| 2020 | November | 546 | 1574.16512 | Warning |
| 2020 | December | 19 | 105.79385 | No Warning |
| 2021 | January | 32 | 32.87859 | No Warning |
| 2021 | February | 9 | 11.27687 | No Warning |
| 2021 | March | 13 | 19.45207 | No Warning |
| 2021 | April | 3 | 13.18208 | No Warning |
| 2021 | May | 43 | 30.71929 | No Warning |
| 2021 | June | 272 | 60.45682 | No Warning |
| 2021 | July | 2286 | 104.77024 | No Warning |
| 2021 | August | 7698 | 894.30073 | No Warning |
| 2021 | September | 7841 | 1063.55852 | Warning |
| 2021 | October | 5458 | 537.42033 | No Warning |
| 2021 | November | 3567 | 192.76543 | No Warning |
| 2021 | December | 1207 | 51.74877 | No Warning |

**Table S4.** Predicted dengue cases with warning status by XGBoost model (2022-2026)

| **Year** | **Month name** | **Predicted** | **Warning** |
| --- | --- | --- | --- |
| 2022 | January | 5.602995 | No Warning |
| 2022 | February | 5.649109 | No Warning |
| 2022 | March | 5.656142 | No Warning |
| 2022 | April | 5.656142 | No Warning |
| 2022 | May | 5.677825 | No Warning |
| 2022 | June | 18.151812 | Warning |
| 2022 | July | 47.244173 | Warning |
| 2022 | August | 55.456242 | Warning |
| 2022 | September | 52.124267 | Warning |
| 2022 | October | 51.693838 | Warning |
| 2022 | November | 48.301357 | Warning |
| 2022 | December | 46.895653 | Warning |
| 2023 | January | 5.602995 | No Warning |
| 2023 | February | 5.649109 | No Warning |
| 2023 | March | 5.656142 | No Warning |
| 2023 | April | 5.656142 | No Warning |
| 2023 | May | 5.677825 | No Warning |
| 2023 | June | 18.151812 | Warning |
| 2023 | July | 47.244173 | Warning |
| 2023 | August | 55.456242 | Warning |
| 2023 | September | 52.124267 | Warning |
| 2023 | October | 51.693838 | Warning |
| 2023 | November | 48.301357 | Warning |
| 2023 | December | 46.895653 | Warning |
| 2024 | January | 5.602995 | No Warning |
| 2024 | February | 5.649109 | No Warning |
| 2024 | March | 5.656142 | No Warning |
| 2024 | April | 5.656142 | No Warning |
| 2024 | May | 5.677825 | No Warning |
| 2024 | June | 18.151812 | Warning |
| 2024 | July | 47.244173 | Warning |
| 2024 | August | 55.456242 | Warning |
| 2024 | September | 52.124267 | Warning |
| 2024 | October | 51.693838 | Warning |
| 2024 | November | 48.301357 | Warning |
| 2024 | December | 46.895653 | Warning |
| 2025 | January | 5.602995 | No Warning |
| 2025 | February | 5.649109 | No Warning |
| 2025 | March | 5.656142 | No Warning |
| 2025 | April | 5.656142 | No Warning |
| 2025 | May | 5.677825 | No Warning |
| 2025 | June | 18.151812 | Warning |
| 2025 | July | 47.244173 | Warning |
| 2025 | August | 55.456242 | Warning |
| 2025 | September | 52.124267 | Warning |
| 2025 | October | 51.693838 | Warning |
| 2025 | November | 48.301357 | Warning |
| 2025 | December | 46.895653 | Warning |
| 2026 | January | 5.602995 | No Warning |
| 2026 | February | 5.649109 | No Warning |
| 2026 | March | 5.656142 | No Warning |
| 2026 | April | 5.656142 | No Warning |
| 2026 | May | 5.677825 | No Warning |
| 2026 | June | 18.151812 | Warning |
| 2026 | July | 47.244173 | Warning |
| 2026 | August | 55.456242 | Warning |
| 2026 | September | 52.124267 | Warning |
| 2026 | October | 51.693838 | Warning |
| 2026 | November | 48.301357 | Warning |
| 2026 | December | 46.895653 | Warning |

**Table S5.** monthly actual and predicted dengue cases with warning status by LightGBM model (2019-2021)

| **Year** | **Month name** | **Actual** | **Predicted** | **Warning** |
| --- | --- | --- | --- | --- |
| 2019 | January | 38 | 10.596460 | No Warning |
| 2019 | February | 18 | 14.424771 | No Warning |
| 2019 | March | 17 | 15.992998 | No Warning |
| 2019 | April | 58 | 15.714580 | No Warning |
| 2019 | May | 193 | 42.783466 | No Warning |
| 2019 | June | 1884 | 110.878252 | No Warning |
| 2019 | July | 16253 | 391.312696 | No Warning |
| 2019 | August | 52636 | 398.306635 | No Warning |
| 2019 | September | 16856 | 678.999959 | Warning |
| 2019 | October | 8113 | 902.599480 | Warning |
| 2019 | November | 4011 | 514.368442 | Warning |
| 2019 | December | 1247 | 475.926863 | Warning |
| 2020 | January | 111 | 65.895460 | No Warning |
| 2020 | February | 45 | 11.555458 | No Warning |
| 2020 | March | 27 | 15.668049 | No Warning |
| 2020 | April | 25 | 26.601254 | No Warning |
| 2020 | May | 10 | 42.783466 | No Warning |
| 2020 | June | 23 | 122.554103 | No Warning |
| 2020 | July | 68 | 497.759886 | Warning |
| 2020 | August | 163 | 359.744619 | No Warning |
| 2020 | September | 47 | 462.220743 | Warning |
| 2020 | October | 109 | 678.999959 | Warning |
| 2020 | November | 546 | 475.926863 | Warning |
| 2020 | December | 19 | 355.982428 | No Warning |
| 2021 | January | 32 | 27.208721 | No Warning |
| 2021 | February | 9 | 6.718803 | No Warning |
| 2021 | March | 13 | 9.154965 | No Warning |
| 2021 | April | 3 | 8.782685 | No Warning |
| 2021 | May | 43 | 16.306711 | No Warning |
| 2021 | June | 272 | 117.970750 | No Warning |
| 2021 | July | 2286 | 195.699792 | No Warning |
| 2021 | August | 7698 | 318.382631 | No Warning |
| 2021 | September | 7841 | 379.654157 | No Warning |
| 2021 | October | 5458 | 493.900685 | Warning |
| 2021 | November | 3567 | 328.070992 | No Warning |
| 2021 | December | 1207 | 303.525430 | No Warning |

**Table S6.** Predicted dengue cases with warning status by LightGBM model (2022-2026)

| **Year** | **Month Name** | **Predicted** | **Warning** |
| --- | --- | --- | --- |
| 2022 | January | 11.67451 | No Warning |
| 2022 | February | 11.67451 | No Warning |
| 2022 | March | 11.67451 | No Warning |
| 2022 | April | 11.67451 | No Warning |
| 2022 | May | 11.67451 | No Warning |
| 2022 | June | 24.45516 | No Warning |
| 2022 | July | 187.66272 | No Warning |
| 2022 | August | 170.54432 | No Warning |
| 2022 | September | 170.54432 | No Warning |
| 2022 | October | 191.17489 | Warning |
| 2022 | November | 191.17489 | Warning |
| 2022 | December | 191.17489 | Warning |
| 2023 | January | 11.67451 | No Warning |
| 2023 | February | 11.67451 | No Warning |
| 2023 | March | 11.67451 | No Warning |
| 2023 | April | 11.67451 | No Warning |
| 2023 | May | 11.67451 | No Warning |
| 2023 | June | 24.45516 | No Warning |
| 2023 | July | 187.66272 | No Warning |
| 2023 | August | 170.54432 | No Warning |
| 2023 | September | 170.54432 | No Warning |
| 2023 | October | 191.17489 | Warning |
| 2023 | November | 191.17489 | Warning |
| 2023 | December | 191.17489 | Warning |
| 2024 | January | 11.67451 | No Warning |
| 2024 | February | 11.67451 | No Warning |
| 2024 | March | 11.67451 | No Warning |
| 2024 | April | 11.67451 | No Warning |
| 2024 | May | 11.67451 | No Warning |
| 2024 | June | 24.45516 | No Warning |
| 2024 | July | 187.66272 | No Warning |
| 2024 | August | 170.54432 | No Warning |
| 2024 | September | 170.54432 | No Warning |
| 2024 | October | 191.17489 | Warning |
| 2024 | November | 191.17489 | Warning |
| 2024 | December | 191.17489 | Warning |
| 2025 | January | 11.67451 | No Warning |
| 2025 | February | 11.67451 | No Warning |
| 2025 | March | 11.67451 | No Warning |
| 2025 | April | 11.67451 | No Warning |
| 2025 | May | 11.67451 | No Warning |
| 2025 | June | 24.45516 | No Warning |
| 2025 | July | 187.66272 | No Warning |
| 2025 | August | 170.54432 | No Warning |
| 2025 | September | 170.54432 | No Warning |
| 2025 | October | 191.17489 | Warning |
| 2025 | November | 191.17489 | Warning |
| 2025 | December | 191.17489 | Warning |
| 2026 | January | 11.67451 | No Warning |
| 2026 | February | 11.67451 | No Warning |
| 2026 | March | 11.67451 | No Warning |
| 2026 | April | 11.67451 | No Warning |
| 2026 | May | 11.67451 | No Warning |
| 2026 | June | 24.45516 | No Warning |
| 2026 | July | 187.66272 | No Warning |
| 2026 | August | 170.54432 | No Warning |
| 2026 | September | 170.54432 | No Warning |
| 2026 | October | 191.17489 | Warning |
| 2026 | November | 191.17489 | Warning |
| 2026 | December | 191.17489 | Warning |

**Table S7.** Model performance comparison

| **Model** | **MAE** | **RMSE** | **R²** |
| --- | --- | --- | --- |
| **RF Model** | 1.599512 | 1.976182 | 0.416075 |
| **XGBoost Model** | 1.680886 | 1.954354 | 0.4289033 |
| **LightGBM Model** | 1.599512 | 1.976182 | 0.416075 |

RF: Random Forest; XGBoost: eXtreme Gradient Boosting; LightGBM: Light Gradient Boosting Machine; MAE: Mean absolute error; RMSE: Root mean square error; R²: R-squared.

**Table S8.** Mean SHAP values for features in LightGBM model (Climate, Socio-demographic, and landscape variables)

| **Factor** | **Variable** | **Description** | **Mean SHAP Value** |
| --- | --- | --- | --- |
| Climate | x2 | Minimum temperature (°C) | 0.938 |
|  | x4 | Relative humidity (%) | 0.766 |
|  | x1 | Mean temperature (°C) | 0.718 |
|  | x3 | Maximum temperature (°C) | 0.715 |
|  | x7 | Wind Speed at 50 Meters Maximum (m/s) | 0.650 |
|  | x6 | Surface Pressure (kPa) | 0.612 |
|  | x5 | Rainfall (mm) | 0.607 |
| Socio-demographic | x8 | Population density | 0.658 |
|  | x14 | Access to electricity (% of population) | 0.299 |
|  | x18 | Current health expenditure | 0.290 |
|  | x12 | Adult literacy rate (% of population) | 0.120 |
|  | x13 | Total unemployment (% of total labor force) | 0.0796 |
|  | x17 | Air transport, Passengers Carried | 0.0312 |
|  | x16 | Population Growth | 0.00642 |
|  | x10 | Gross National Income (K US$) | 0 |
|  | x11 | Poverty head-count ratio (% of population) | 0 |
|  | x15 | Safe sanitation service (% of population) | 0 |
|  | x9 | Gross Domestic Product (Billion US$) | 0 |
| Landscape | x20 | Agricultural land (% of total land area) | 0.730 |
|  | x19 | Forest area (% of total land area) | 0.419 |
|  | x21 | Arable Land (% of the total land area) | 0.0850 |
|  | x22 | Waste collection (tons) | 0.000000191 |
